# Correlation Analysis between Obstructive Sleep Apnea Syndrome (OSAS) and Heart Rate Variability

**Published:** 2017-11

**Authors:** Jiayong XIE, Wenjuan YU, Zongren WAN, Fei HAN, Qiaojun WANG, Rui CHEN

**Affiliations:** 1. Sleeping Center, Second Affiliated Hospital of Soochow University, Suzhou, PR China; 2. Xinghua People’s Hospital, Xinghua, PR China; 3. The Second Affiliated Hospital of Nanjing Medical University, Nanjing, PR China

**Keywords:** OSAS, Cardiac arrhythmias, Electrocardiogram, Polysomnography

## Abstract

**Background:**

Heart rate variability (HRV) represents the sympathetic nervous system activity induced by apnea or hypopnea events among OSAS patients. However, few studies have been conducted to clarify the association between HRV parameters and polysomnography (PSG) diagnostic indices. In our study, we postulate that the prevalence of cardiac arrhythmias is associated with OSAS, and HRV parameters may be an effective method for OSAS screening.

**Methods:**

Overall, 168 participants had been collected from 2011 to 2016 in the Second Affiliated Hospital of Soochow University. By apnea-hypopnea index (AHI), patients were separated into three subsets: AHI < 5 as control group, 5≤AHI<30 as mild-moderate OSAS group and AHI≥30as severe OSAS group. HRV and PSG parameters were collected based on electrocardiography and polysomnography system. Correlation analyses between standard deviation of R-R intervals (SDNN), SDNN index, RMSSD, PNN50, low frequency (LF), high frequency (HF) and LF/HF ratio and the AHI, ODI and MI were performed by Spearman’s correlation analysis.

**Results:**

Compared with control group (64.5%) or mild-moderate OSAS group (67.3%), the prevalence of arrhythmias was considerably greater in severe OSAS group (*P*<0.05). Moreover, we demonstrated that LF/HF was greater in two OSAS groups than the normal group.

**Conclusion:**

Correlation analyses revealed a significant and positive relation between the LF/HF and AHI, ODI and MI in OSAS patients. Severe OSAS could be attributed to enhanced danger of incident arrhythmia. LF/HF ratio as a relevant feature may be an effective parameter for detecting OSAS.

## Introduction

Obstructive sleep apnea syndrome (OSAS) has been increasingly recognized as a complex and heterogeneous disorder, which features too much daytime sleepiness, tumultuous snoring, recurrent occurrences of upper airway obstruction during sleep and nocturnal hypoxemia (1, 2). OSAS has a prevalence of 13% in adult men and 6% in adult women and forebodes unconditionally numerous illnesses (3, 4). Severe OSAS is associated with greater risk of incident hypertension(5), diabetes (6), obesity(7)and cardiac arrhythmias (8). Moreover, numerous disordered events, including apneas, hypopneas and micro-arousals, occur during the OSAS procession (4).

Therefore, it is important to monitor OSAS, which is beneficial to cardiovascular diseases (CVDs) prevention (9, 10). Recently, there was a correlation between OSAS and HF, and the OSAS prevalence rate was from 21% to 82% in patients with HF (11–13).Cardiac arrhythmiascan foretells a number of cardiac illnesses(14). Therefore, in-depth studies regarding the association of OSAS and cardiac arrhythmias are quite meaningful to CVDs diagnosis.

OSAS was an independent risk factor of various CVDs, including cardiac arrhythmias (15, 16). Electrocardiogram (ECG)is often used for cardiac arrhythmias screening in clinical practices, but screening for OSAS is uncommon, because unequivocal diagnosis usually involves spending a night in asleep laboratory and many physiological variables are uninterruptedly recorded by full-night polysomnography (PSG) (17, 18). Although cardiac arrhythmias occur more often in patients with OSAS, we do not know if OSAS is a primary etiological factor for cardiac arrhythmias occurrence. The prevalence of arrhythmias is significantly increased in subjects with sleep-disordered breathing as compared to those without at night (19, 20). Moreover, the hemodynamic, autonomic changes, atrial and ventricular electrical and structural changes caused by OSAS promote bradyarrhythmias, atrial fibrillation and ventricular arrhythmias, which may contribute to increased risk of CVDs (21, 22). Therefore, increasing awareness of the correlation between OSAS and cardiac arrhythmias plays a crucial role in reducing morbidity and mortality of patients with CVDs.

In the present study, we explored the associations between OSAS and cardiac arrhythmias. Holter ECG recordings and assorted PSG diagnostic indices (including the AHI, MI and ODI) were collected, while the linear models were constructed to describe them.

## Materials and Methods

### Participants

Altogether 168 participants had been collected from January 2011 to December 2016 in the Department of the Second Affiliated Hospital of Soochow University (Suzhou, China). We carried out a retrospective observational study in three groups: A total of 168 participants met the inclusion criteria divided into three groups. Thirty-one of 168 (18.5%) with AHI less than 5 (2.03 ± 1.89) in control group, 5≤AHI<30 (n = 55) as mild-moderate OSAS group and AHI≥30 (n = 82) as severe OSAS group, in view of sleep apnea-hypopnea index (AHI) categories. Height and weight were calibrated to compute the body mass indice (BMI) for all participants.

Clinical experiments were obtained with formal permission from all patients. Our research has been approved by the Ethics Committee of the Second Affiliated Hospital of Soochow University (Suzhou, China).

### Polysomnography (PSG) Data Collection

Polysomnographic recording was performed using Alice 6 (Philips, Netherlands). All of participants underwent seven-hour nighttime monitoring with PSG in bed. No ingest alcohol or caffeine, nap or engage and protracted or tedious exercises, and so on were informed during the study. Apnea could be viewed as being without respiration for more than 10 seconds. Hypopnea could be viewed as the deduction of at least 50% ventilation causing a drop in arterial saturation of at least 4%. OSAS could be viewed as apnea or hypopnea happening at least five times per hour, persisting for more than 10 seconds. The AHI documents the number of apnea-plus-hypopnea incidents every hour during sleep. The ODI assesses the average number of oxygen desaturation incidents every hour during sleep. The MI was computed using the average value of the micro-arousals associated with respiratory incidents each hour during sleep, as previously described (4). We set an AHI< 5/hour as the threshold. We set mild-moderate OSAS to be 5 ≤AHI<30/hour and set severe OSAS to be AHI≥30/hour.

### Electrocardiogram (ECG) Data Collection

From the polysomnographic data, we got electrocardiographic signals and studied them in accordance with Task Force of the European Society of Cardiology and the North American Society of Pacing and Electrophysiology. We classified the parameters of HRV into three domains: time domain includes SDNN, standard deviation of all NN; RMSSD, the square root of the mean of the sum of the squares of differences between adjacent NN intervals; PNN50, NN50 number divided by the sum of all NN intervals; LF, power in low frequency spectrum (0.04–0.15 Hz); HF, power in high frequency spectrum (0.15–0.4 Hz).

### Statistical Analysis

All statistical analyses were carried out with SPSS 18.0 software (SPSS, Inc., Chicago, IL, USA). Statistical differences between two groups were determined with Student’s *t* test. The correlation of HRV parameters and PSG diagnostic indices was analyzed with linear regression analysis. The statistical diagrams were carried out with Graph-Pad Prism software, version 7.0 (GraphPad Software, Inc., La Jolla, CA, USA). We studied groups with one-way analysis of variance, then with Tukey’s multiple comparison test as a post hoc test to study the mean values of each group. *P*< 0.05 represented a statistically notable difference.

## Results

### General clinical characteristics

The clinical characteristics are shown in Table 1. We found few notable differences regarding age and gender among three groups.

**Table 1:** General clinical characteristics of the enrolled participants

***Characteristics***	***Severe OSAS (AHI ≥30)***	***Mild-moderate OSAS (30>AHI ≥ 5)***	***Control (AHI < 5)***
	(n = 82)	(n = 55)	(n = 31)
Age (Years)	50.60 ± 12.28	50.57 ±12.02	48.00±13.24
Gender (male, %)	68(82.9%)	43(78.2%)	27(87.1%)
BMI (kg/m^2^)	28.77 ± 4.03^a,b^	26.63 ± 2.98	24.49± 3.80
Pre-PSG			
Systolic blood pressure (mm Hg)	129.50 ± 15.56	128.39 ± 16.18	127.16 ± 19.65
Diastolic blood pressure (mm Hg)	84.37 ± 12.58	83.06 ± 10.40	83.16 ± 10.02
Post-PSG			
Systolic blood pressure (mm Hg)	133.17 ± 15.00 ^a,b^	127.57 ± 17.16	123.50 ± 16.50
Diastolic blood pressure (mm Hg)	90.80 ± 11.81^a,b^	86.37 ± 9.12	81.41 ± 9.05
Cardiovascular disease risk factors			
Hypertension (%)	47(57.3%)^a^	23(41.8%)	9(29.0%)
Diabetes mellitus (%)	11(13.4%)	7(12.7%)	0
Hepatopathy (%)	40(48.8%)	29(52.7%)	6 (19.4%)
≥ 10 years smoking history (%)	33(40.2%)	21(38.2%)	4(12.9%)
≥ 10 years drinking history (%)	20(24.4%)	16(29.1%)	3(9.7%)
Cardiovascular disease manifestations			
Coronary heart disease (%)	3(3.7%)	3(5.5%)	0
Ischemicstroke (%)	5(6.1%)	6(10.9%)	0
Arrhythmia (%)	73(89.0%)^a,b^	37(67.3%)	20(64.5%)
Ventricular premature beat (%)	47(57.3%)	25(45.5%)	16(51.6%)
Ventricular tachycardia (%)	3(3.7%)	2(3.6%)	0
Atrial premature beat (%)	62(75.6%) ^a,b^	29 (52.7%)	15(48.4%)
Atrial tachycardia (%)	19(23.2%)	7(12.7%)	2(6.5%)
Sinus arrest (%)	4(4.9%)	3(5.5%)	1(3.2%)
Atrioventricular block (%)	7(8.5%)	2(3.6%)	0

a Compared with Control group, P<0.05;

b Compared with Mild-moderate OSAS group, P<0.05.

However, those patients in two OSAS group possessed a higher BMI than those in control group, and intriguingly AHI was positively linked to BMI (*P*<0.001, r=0.466). There was no significant difference of the systolic blood pressure (SBP) and diastolic one (DBP) between Control and OSAS groups before nocturnal PSG diagnosis, while SBP and DBP significantly increased after 7 hours PSG monitoring in severe OSAS group. As expected, regarding the PSG diagnostic indices, including AHI, ODI, MI, STL 90% and SaO_2_, results revealed significant difference between control group and severe OSAS group.

Severe OSAS group experienced a notably higher prevalence of hypertension than control group (57.3% *vs.*29.0%; *P*<0.05). However, little notable difference existed between severe and mild-moderate groups or between mild-moderate and control groups. Regarding rates of diabetes mellitus, hepatopathy, smoking history, coronary heart disease and ischemic stroke, no statistical differences were observed, between groups. Importantly, the prevalence of arrhythmia was markedly increased in OSAS groups and was closely related with the severity of OSAS (Table 1).

### PSG diagnostic indices and HRV parameters

As shown in Table 2, PSG diagnostic indices of three groups varied by the level of OSAS. The heart rate variability (HRV) parameters, including SDNN, SDNN index, RMSSD, PNN50, LF, HF and LF/HF, 5 of 7 parameters showed no statistical significance between groups, while LF/HF appeared higher in the OSAS groups than the control group (Table 3).

**Table 2:** PSG characteristics of the different 3 groups

***PSG characteristics***	***Severe OSAS (AHI ≥30)***	***Mild-moderate OSAS (30>AHI ≥ 5)***	***Control (AHI < 5)***
	
	(n = 82)	(n = 55)	(n = 31)
ODI	40.92 ± 27.96^a,b^	12.66 ± 8.87	2.15 ± 2.26
AHI	59.30 ± 16.24^a,b^	17.55 ± 7.12	2.03 ± 1.89
STL90% (%)	22.67 ± 18.18^a,b^	2.64 ± 5.74	1.54 ± 3.35
SaO_2_ (%)	66.37 ± 15.69^a,b^	79.54 ± 11.09	84.75 ± 14.55
MI	26.09 ± 22.62^a,b^	10.77± 15.38	1.69 ± 5.79

ODI: Oxygen desaturation index; AHI: Apnea hyponea index; STL90%: The percent of time in saturation lower 90%; SaO_2_: The lowest blood oxygen saturation; MI: Micro-arousal index.

(^a^Compared with Control group, P<0.05; ^b^Compared with Mild-moderate OSAS group, P<0.05).

**Table 3:** Comparison of Heart rate variability parameters between OSAS and control groups

	***OSAHS***	***Control***	***P-value***
	(n = 137)	(n = 31)	
SDNN (ms)	125.0 ± 28.4	139.7 ± 25.1	0.011
SDNN index (ms)	54.8 ± 17.7	53.9 ± 18.1	0.794
RMSSD (ms)	26.7 ± 9.5	27.9 ± 11.2	0.566
PNN50 (%)	8.3 ± 7.5	8.6 ± 7.8	0.824
LF (ms^2^)	612.8 ± 321.7	504.8 ± 260.5	0.094
HF (ms^2^)	256.9 ± 175.8	283.7 ± 197.5	0.475
LF/HF (ms^2^/ms^2^)	3.24 ± 2.89	2.28 ± 1.24	0.044

SDNN, standard deviation of all NN; RMSSD, the square root of the mean of the sum of the squares of differences between adjacent NN intervals; PNN50, NN50 count divided by the total number of all NN intervals; LF, power in low frequency range (0.04–0.15 Hz); HF, power in high frequency range (0.15–0.4 Hz).

### Correlation analysis between AHI and the HRV parameters

**Fig. 1:**
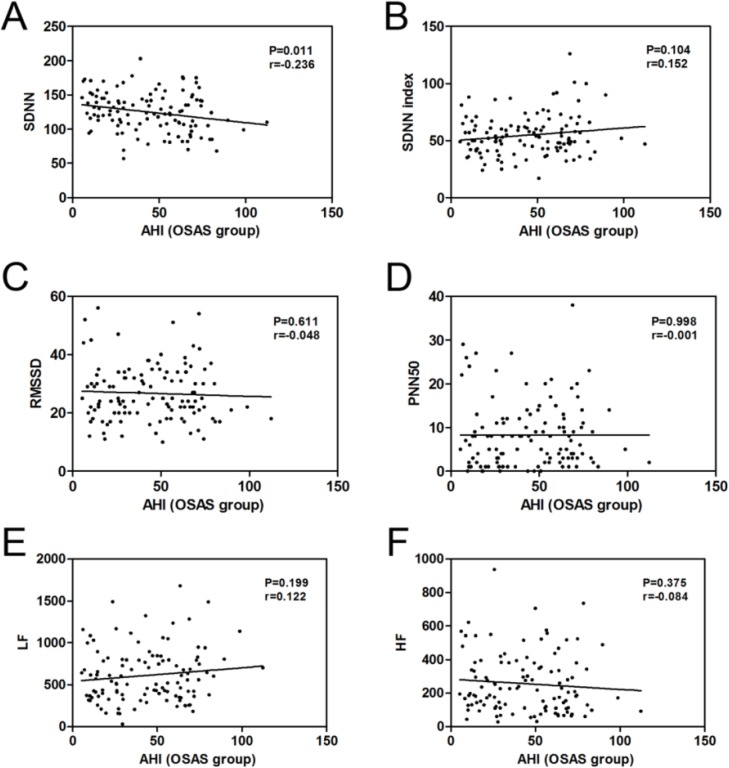
The correlation between the AHI and SDNN (A), SDNN index (B), RMSSD (C), PNN50 (D), LF (E) and HF (F) were performed by Spearman’s correlation analysis in OSAS patients

Correlation coefficients between the HRV parameters and the AHI, ODI and MI were performed in the OSAS group. The results demonstrated that the correlations of AHI and the HRV parameters had not statistical significance (Fig. 1BF) except for the negative correlation between the AHI and SDNN (r = −0.236, *P* = 0.011) among the patients with OSAS (Table 4 and Fig. 1A).

**Table 4:** Correlation between the heart rate variability parameters and AHI, ODI and MI in patients with OSAS

***Parameters***	***AHI***		***ODI***		***MI***	
	***P***	***r***	***P***	***r***	***P***	***r***
SDNN	0.011	−0.236	0.015	−0.226	0.017	−0.226
SDNN index	0.104	0.162	0.305	0.096	0.301	0.099
RMSSD	0.611	−0.048	0.018	−0.219	0.092	−0.161
PNN50	0.998	−0.001	0.515	−0.061	0.373	−0.085
LF	0.199	0.122	0.021	0.281	0.041	0.197
HF	0.375	−0.084	0.098	−0.157	0.183	−0.129

### Correlation analysis between ODI and the HRV parameters

**Fig. 2:**
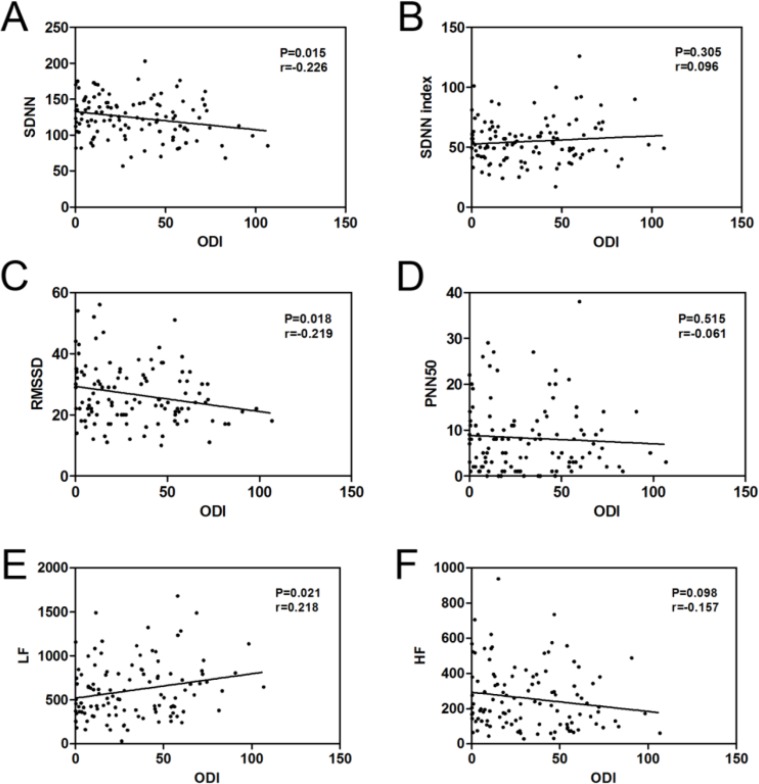
The correlation between the ODI and SDNN (A), SDNN index (B), RMSSD (C), PNN50 (D), LF (E) and HF (F) were performed by Spearman’s correlation analysis in OSAS patients

The ODI was negatively linked to SDNN (r = −0.226, *P* = 0.015, Table 4 and Fig. 2A) and RMSSD (r = −0.219, *P* = 0.018, Table 4 and Fig. 2C) and was positively linked to LF (r = 0.218, *P* = 0.021, Table 4 and Fig. 2E). No notable connections were observed between ODI and SDNN index, PNN50 and HF (Table 4 and Fig. 2B, 2D, 2F).

### Correlation analysis between MI and the HRV parameters

**Fig. 3:**
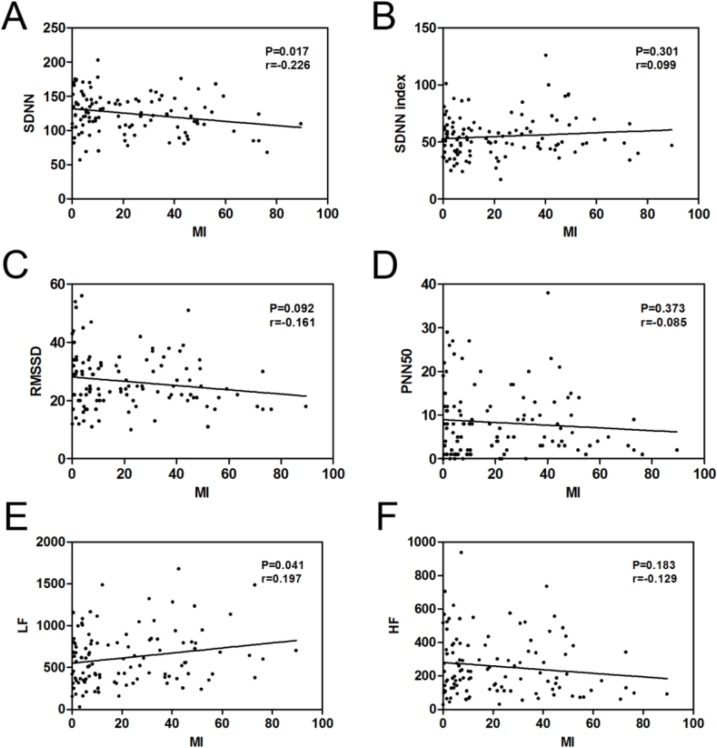
The correlation between the MI and SDNN (A), SDNN index (B), RMSSD (C), PNN50 (D), LF (E) and HF (F) were performed by Spearman’s correlation analysis in OSAS patients

The MI was negatively linked to SDNN (r = −0.226, *P* = 0.017, Table 4 and Fig. 3A) and was positively linked to LF (r = 0.197, *P* = 0.041, Table 4 and Fig. 3E). No significant correlations were observed between MI and SDNN index, RMSSD, PNN50 and HF (Table 4 and Fig. 3B–D, 3F).

### Correlation analyses between LF/HF and AHI, ODI and MI

**Fig. 4:**
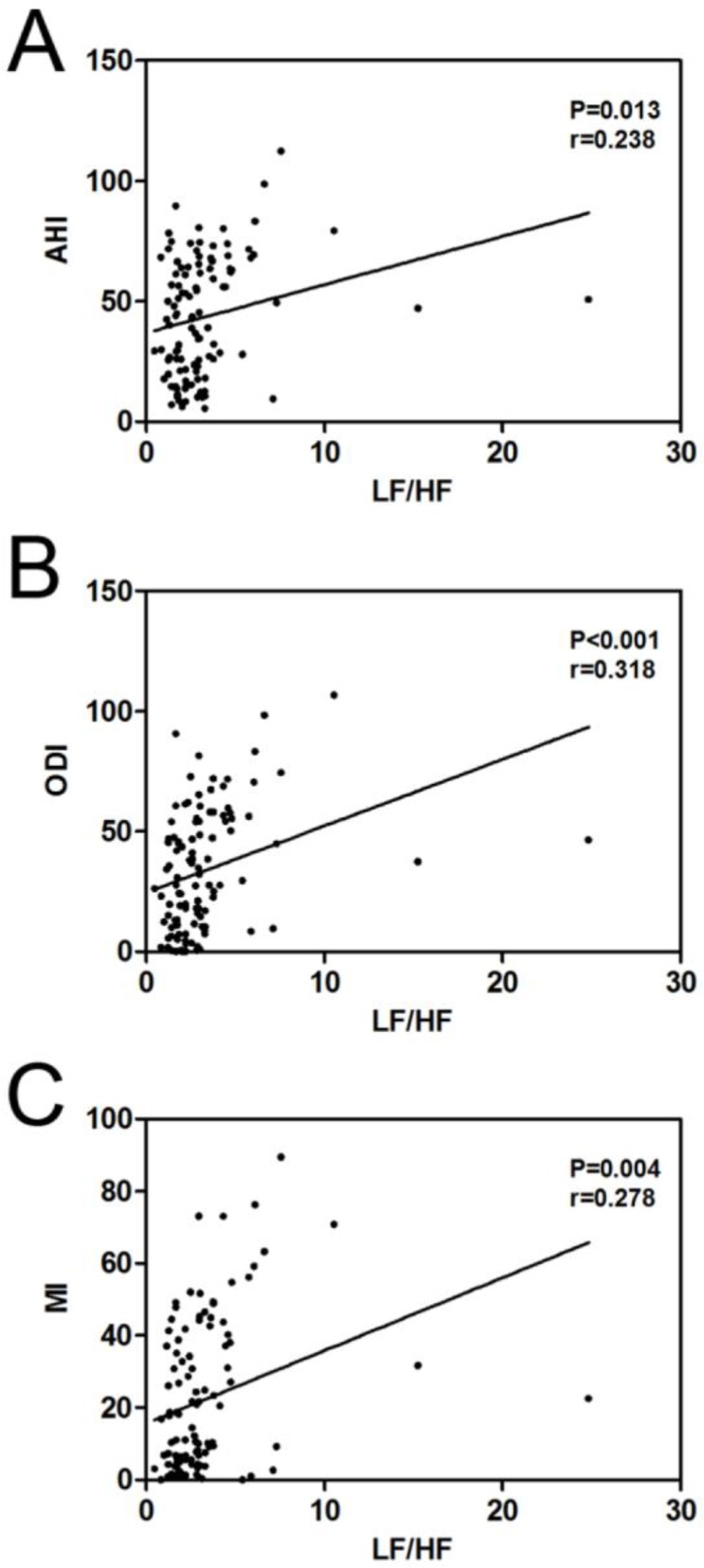
The correlation between the LF/HF and AHI (A), ODI (B) and MI (C) were performed by Spearman’s correlation analysis in OSAS patients

The LF/HF was notably and positively linked to AHI (r = 0.238, *P* = 0.013, Fig. 4A), ODI (r = 0.318, *P*< 0.001, Fig. 4B) and MI (r = 0.278, *P* = 0.004, Fig. 4C) in OSAS patients.

## Discussion

In this study, our results demonstrated that severe OSAS was associated with greater risk of incident arrhythmia. In correlation analysis between AHI and HRV parameters in OSAS patients, only SDNN was appeared negatively linked to AHI. Moreover, AHI, ODI and MI were only positively correlated with LF/HF. The HRV parameters showed little notable difference between OSAS group and control group except SDNN and LF/HF. These findings suggest that ECG, as a noninvasive, simple and affordable method, may not be an effective method for OSAS screening. However, LF/HF as a HRV parameter provides theoretical and practical basis for OSAS screening.

OSAS was closely related to the chronic diseases, especially diseases involving cardiovascular system (4, 19, 23). OSAS is linked to increased arrhythmogenic risk by provoking periodic elevations in sympathetic activity and parasympathetic withdrawal, which might later cause tachycardia, peripheral vasoconstriction, increased blood pressure and myocardial oxygen demand (10). Compared to participants with AHI < 5, patients having serious sleep-disordered breathing (SDB, AHI ≥ 15) have nearly three-fold unadapted possibility of any cardiac arrhythmia, and SDB is independently linked to night time cardiac arrhythmias (24). Increasing severity of SDB is linked to a gradual growth in possibility of atrial fibrillation and complex ventricular ectopic in a large cohort of older men (25). In consistent with several clinic-based studies (23–25), our findings indicated that growing level of AHI was linked to an increasing risk of incident arrhythmia. Treatment of OSAS patients with cardiac arrhythmias, the principal therapy, continuous positive airway pressure (CPAP), is refractory (15). The distributed myocardial fibrosis is identified as the underlying molecular mechanisms for reentrant ventricular tachyarrhythmias (10). Simultaneously, acute and chronic intermittent hypoxia can be pro-arrhythmogenic by inducing reactive oxygen species (ROS) and increasing sympathetic nerve activity (24, 26, 27).These results suggest that early diagnosis and therapy of OSAS are important to reduce the incidence of arrhythmia and sudden death.

HRV means the time-variation between consecutive heartbeats can reflect state of the autonomic nervous system (ANS) inpatients with CVD (23, 28). Thus, it is a favorite monitoring method to investigate the cardiac autonomic function (29). Moreover, HRV parameters are often employed to study OSAS, and the SDNN index and RMSSD are feasible forecasters of OSAHS screening (4,30,31). SDNN is significantly increased and positively correlated with PSG diagnostic indices in OSAS patients compared with healthy control (4, 23). On the contrary, SDNN was significantly decreased and negatively correlated with AHI (*P* = 0.011, r = −0.236), ODI (*P* = 0.015, r = −0.226) and MI (*P* = 0.017, r = −0.226) in OSAS patients as compared to control group. The HRV parameters are highly linked to the PSG diagnostic indices, AHI, ODI and MI, among OSAS participants and suggests that HRV parameters may be the powerful tools for OSAS screening in place of PSG monitoring (4). However, using laboratory-based methods to evaluate OSAS combined with PSG monitoring and ECG examination, we observed little notable difference link between HRV parameters and PSG diagnostic indices in OSAS patients.

In particular, the LF/HF ratio is considered as the most helpful parameter to determine the level of AHI in OSAS patients (32). The similar results were confirmed by Flevari et al (31). The LF band, ranging from 0.04 Hz to 0.15 Hz, is linked to sympathetic activity or sympathetic and para-sympathetic activity, whereas the HF band, ranging from 0.15 to 0.4 Hz, is principally related to parasympathetic activity . Usually, the LF/HF ratio is denotes the sympathovagal balance, which has been interestingly conducted in OSAS (23). Our research showed that LF/HF ratio was greatly enhanced in OSAS group according to control group, and LF/HF ratio was markedly and positively linked to PSG parameters, including AHI, ODI and MI, in OSAS group. Therefore, we conclude that LF/HF ratio may be suitable for screening OSAS in patients unable to take conventional PSG monitoring.

Lastly, the limitations of this research are pointed out. Firstly, limitation of the small sample size may be a possible reason for the controversial conclusions. Moreover, this is a cross-sectional study design; therefore, we cannot make any certain conclusion about the correlation between HRV parameters and PSG diagnostic indices. Moreover, we must emphasize that all of the correlation coefficient found in our population are only weak (r<0.3). Thus, correlation between HRV parameters and PSG diagnostic indices in OSAS patients is complicated in clinical practice.

## Conclusion

Severe OSAS was associated with high morbidity of the patients with arrhythmia. However, ECG examination may not be an effective method for OSAS screening in place of PSG monitoring. Our study also offered the possibility that, the potential use of LF/HF ratio as a relevant feature that can be involved in detecting OSAS, and our findings should be verified by a much larger group of participants.

## Ethical considerations

Ethical issues (Including plagiarism, informed consent, misconduct, data fabrication and/or falsification, double publication and/or submission, redundancy, etc.) have been completely observed by the authors.
